# Awareness of Palliative Care Among Diploma Nursing Students

**DOI:** 10.4103/0973-1075.78445

**Published:** 2011

**Authors:** Suja Karkada, Baby S Nayak, Malathi

**Affiliations:** Department of Community Health Nursing, Manipal College of Nursing, Manipal University, Manipal, Udupi Dist., Karnataka, India; 1 Department of Child Health Nursing, Manipal College of Nursing, Manipal University, Manipal, Udupi Dist., Karnataka, India

**Keywords:** Attitude, Knowledge, Palliative care, Practice

## Abstract

**Background:**

The goal of palliative care is not to cure, but to provide comfort and maintain the highest possible quality of life for as long as life remains. The knowledge of nurses influences the quality of care provided to these patients. The present study aimed at identifying the level of knowledge and attitude of nursing students who are the future caretakers of patients, which helps to make recommendations in incorporating palliative care concepts in the nursing curriculum.

**Objectives:**

(1) To assess the level of knowledge of nursing students on palliative care; (2) To identify the attitude of nursing students towards palliative care; (3) To find the correlation between the knowledge and attitude of nursing students; (4) To find the association between nursing students’ knowledge, attitude and selected demographic variables.

**Materials and Methods:**

A correlative survey was carried out among 83 third-year Diploma Nursing students by using cluster sampling method from selected nursing schools of Udupi district.

**Results:**

The data analyzed showed that the majority (51%) of them was in the age group of 21years and 92% of them were females. Only 43.4% of them were aware of the term palliative care and it was during their training period. The data showed that 79.5% of students had poor knowledge (6.4± 1.64) on palliative care and 92.8% of them had favorable attitude (56.7± 8.5) towards palliative care. The chi-square showed a significant association between knowledge and age (χ^2^=18.52,*P*<0.01) of the nursing students.

**Conclusion:**

Palliative care aspects should be incorporated in the diploma nursing curriculum.

## INTRODUCTION

Palliative care, also called comfort care, is primarily directed at providing relief to a terminally-ill person through symptom management and pain management. The goal is not to cure, but to provide comfort and maintain the highest possible quality of life for as long as life remains. Well-rounded palliative care programs also address mental health and spiritual needs along with the physical needs. The focus is not on death, but on compassionate specialized care for the living. Palliative care is well-suited to an interdisciplinary team that provides support for the whole person and those who are sharing the person’s journey in love.[1–3]

Nurses are the most numerous healthcare providers in almost every country; they are often the primary caregivers. Historically, nurses have been involved in the provision of palliative care. Nurses have played various roles in the development of palliative care, offering leadership, support and focus for the movement.[2,3,4] However, despite this type of support for palliative care, nursing and a continued involvement in palliative care, nursing has lagged behind other disciplines in the development of palliative care nursing education curricula. This underdeveloped educational foundation has contributed to difficulties in defining the role of nurses in palliative care.

### Purpose

The present study aimed at assessing the level of knowledge and attitude of nursing students towards palliative care, which will help to identify the learning needs so that palliative care aspects can be incorporated in their curriculum formally or informally.

## OBJECTIVES

The objectives of the study were to:

1. Assess the level of knowledge of nursing students on palliative care
2. Identify the attitude of nursing students towards palliative care
3. Find the correlation between knowledge and attitude of nursing students
4. Find the association between nursing students’ knowledge, attitude and selected demographic variables

### Hypotheses

The study attempted to test following hypotheses:

(All the hypotheses were tested at 0.05 level of significance.)

- There will be a significant relationship between nursing students’ knowledge and attitudes and selected variables.
- There will be a significant association between nursing students’ knowledge, attitude and selected demographic variables.

### Assumption

The study assumed that

- the nursing students will have some knowledge on palliative care
- a favorable attitude of nursing students will promote better patient care

## MATERIALS AND METHODS

A cross-sectional correlative survey study was conducted among third-year diploma nursing students from selected nursing schools of Udupi district. A cluster sampling technique was used to select 83 third-year diploma nursing students from two training institutions of Udupi District. Sample size was calculated and required sample size was 74. Taking non-response into consideration a sample of 83 were chosen for the study.

### Data collection instruments

Data was collected by using a structured and validated questionnaire. The questionnaire had three parts.

Part 1: The Demographic Proforma consisted of age, gender, life-threatening illness in the family, and sources of information on palliative care.

Part 2: A structured questionnaire consisting of 20 Multiple Choice Questions (MCQs) was developed to assess the knowledge on palliative care. Each correct response carried one mark. Total score was 20. Knowledge scores were categorized into poor (≤7), average (8– 4), good (≥15).

Part 3: Attitude scale – Likert type of scale was developed. It had 18 item rating scale with the highest score of 5 for each option and total possible score was 90. The attitude scores were categorized into favorable (>45) and unfavorable (<45).

Validity of the tools was established by submitting them to three experts and there was 100% agreement on all items. Reliability was established by administering the tool to 20 students. Reliability coefficient of the knowledge questionnaire was established by split-half method using Spearman Brown prophecy formula. Reliability coefficient was found to be 0.9, and reliability coefficient of attitude scale was computed using Chronbach’s alpha and was (α = 0.72).

### Data collection procedure

Administrative permission was obtained from the Principals of selected schools. Verbal consent was obtained from the study participants. Questionnaire was administered to them in the classroom setting. The time taken to respond to the questionnaire was 40–45 min. Data were analyzed using SPSS package Version 11.5. The data was analyzed using descriptive (frequency and percentage) and inferential statistics based on the objectives and hypotheses.

## RESULT

**Figure 1:**
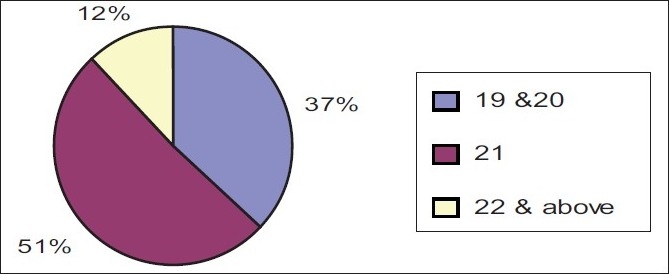
Agewise distribution of sample

**Figure 2:**
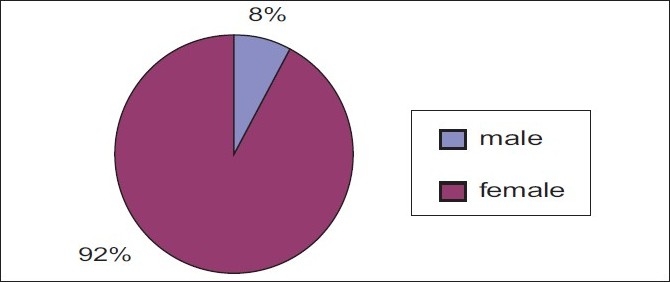
Gender wise distribution of sample

The data presented in Figures 1 and 2 shows that among 83 samples, the majority (51%) of the samples were in the age group of 21years and majority (92%) of them were females.

None of them had a family member with a life-threatening illness. Only 43.4% of them were aware of the term palliative care and it was during learning about cancer management.

The description of knowledge and attitude scores shows that 79.5% of students had poor knowledge on palliative care and 92.8% of them had favorable attitude towards palliative care [Table 1]. The mean and standard deviation of the knowledge and attitude score of the students are shown in Table 2.

**Table 1:** Frequency and percentage distribution of sample based on knowledge and attitude score *n* = 83

Variable	Category	Frequency	Percentage
Knowledge	Poor	66	79.5
	Average	17	20.5
Attitude	Favorable	77	92.8
	Unfavorable	6	7.2

**Table 2:** Mean and Standard deviation of the knowledge and attitude score of the students *n* = 83

Variable	Mean	Standard deviation	Standard error of mean	Median
Knowledge	6.4	1.64	0.18	6
Attitude	56.7	8.5	0.93	57

The Pearson’s correlation coefficient was computed which indicates that there exists a negative correlation between knowledge and attitude scores, which is not significant at 0.05 level of significance [Table 3].

**Table 3:** Pearson correlation coefficient showing the relationship between knowledge and attitude scores *n* = 83

Variable	Mean	Standard deviation	Standard error of mean	Correlation coefficient
Knowledge	6.4	1.64	0.18	- 0.117
Attitude	56.7	8.5	0.93	P = 0.2

The chi-square computed to find the association between knowledge, attitude and selected variables shows a significant association between the knowledge and age of the nursing students. The chi-square shows that there was no significant association between attitude and variables among nursing students [Tables 4 and 5].

**Table 4:** Chi square value computed between knowledge and selected demographic variables *n* = 83

Variable	Knowledge		Chi square	*P* value
Age	Poor	Average		
19 and 20	23	8	18.52	0.01
21	26	6		
22 and above	7	3		
Gender				
Male	7	0	3.37	0.06
Female	59	17		
Awareness of term palliative care				
Yes	32	4	3.61	0.57
No	34	13		

**Table 5:** Chi square value computed between attitude and selected demographic variables *n* = 83

Variable	Attitude		Chi square	*P* value
Age	Favorable	Unfavorable		
19 and 20	27	4		
21	40	2	4.85	0.6
22 and above	10	0		
Gender				
Male	7	0	1.09	0.29
Female	70	6		
Awareness of term palliative care				
Yes	36	0		
No	41	6	7.18	0.07

## CONCLUSION AND DISCUSSION

The majority (79.5%) of students had poor knowledge on palliative care but they had a favorable attitude towards palliative care. There exists a negative correlation between knowledge and attitude scores of nursing students on palliative care. There is a significant association between the age of the nursing students and knowledge regarding palliative care. However, other variables were independent of knowledge. The World Health Organization has called for training institutions to make palliative care compulsory in courses leading to a basic professional qualification[4] while symptom control is extremely important, it cannot be the only component of a palliative care curriculum. Patient - centered communication, ethical issues, decision - making at the end of life, whole person care and interdisciplinary work are important and can have a lasting impact on future health practice[5]. These aspects of palliative care need to be integrated into the entire curriculum. In practice, nurses carry out simple and complex tasks continuously, making their contributions to the multidisciplinary palliative care team central, yet difficult to circumscribe. The competency of nurses is demonstrated by the work they do, that is their contribution to the multidisciplinary pain team and improvements in patients’ pain scores. Competency will only become visible when all places of care adopt and use a consistent international language describing nursing interventions and outcomes, such as time spent with patients, length of patient stay, greater patient satisfaction, and improvements in nursing knowledge. The training and competency of generalist nurses is recognized as the greatest area of need in nursing education. To become competent nursing graduates need to be prepared to take care of the terminally ill patient at the grass root level. They need to do home visits for patients who are ill and wish to be at home in their last stages of life. Home nursing should become part of nursing training which will promote a positive attitude among the nursing students.
